# Targeting iNAMPT and NAD Biosynthesis to Break the Obesity-Associated Liver Cancer Link

**DOI:** 10.3390/biomedicines13071533

**Published:** 2025-06-24

**Authors:** Kelly Thornton, Linda Torres, Elisa L. Pedone, Jessica S. Waltenbaugh, Cassandra M. Swanson, Emily Gonzalez, Ramona S. Price

**Affiliations:** Nutrition and Foods Program, School of Family and Consumer Sciences, College of Applied Arts, Texas State University, San Marcos, TX 78666, USA

**Keywords:** liver cancer, obesity, adipokine, visfatin, NAMPT

## Abstract

**Background and Objectives:** Obesity is linked to liver cancer through metabolic mechanisms and can promote tumor growth through metabolic impairment, decreased lipid metabolism, and interference of the energy balance in the liver. NAMPT is an enzyme expressed in the liver and is involved in the progression of tumors in obesogenic environments, while iNAMPT is known to be the rate-limiting enzyme in the synthesis of NAD, an essential coenzyme involved in ATP synthesis which promotes a pro-growth environment in the context of obesity. Because iNAMPT and cellular energetics, a hallmark of cancer, play an important role in liver cancer progression, it has become a target for cancer therapies focused on inhibiting its functions. The objective of this study was to determine the contribution of NAD biosynthesis in obesity-associated liver cancer progression. **Methods**: Cell culture studies were conducted with serum from male mice randomized to diet-induced obesity (OB) or control (CR) ± FK866 (iNAMPT inhibitor) in SNU, HepG2 human liver cancer cells, and Hepa 1-6 liver murine cells. Protein analysis of pAkt and pErk was performed via immunoblot. Cytotoxicity, reactive oxygen species (ROS), cell viability, and invasion were also measured in the cells. For the mouse model, the C57BL/6J male mice were randomized to the DIO or CR group. At 21 weeks of age, the mice were injected subcutaneously with Hepa 1-6 liver cancer cells. At 23 weeks, the mice received an I.P. injection of FK866 (30 mg/kg) for 2 weeks. The tumor and mouse weights were measured. **Results**: The cells exposed to OB sera showed increased proliferation, lactate dehydrogenase (LDH) secretion, ROS, and invasion. FK866 decreased proliferation, LDH secretion, ROS, and invasion for all liver cancer cells. The cells exposed to CR sera and OB + FK866 resulted in more LDH, suggesting increased apoptosis compared with OB sera. The OB sera increased phosphorylation of Akt, which was suppressed by FK866 compared with the OB group. In liver cancer cells, physiological and cellular signaling is affected differently when inhibiting NAD biosynthesis in an in vitro model of obesity and liver cancer. In vivo, the diet-induced obese (DIO) mice weighed significantly more than the mice fed a control diet. In addition, 70% of the DIO mice developed tumors, compared with 20% of the CR mice, and had tumors with greater volumes and weights. NAD inhibition blocked obesity-induced tumor growth. **Conclusions**: In this study, we demonstrate that inhibition of iNAMPT resulted in suppression of tumor growth in the context of obesity. Identifying pre-clinical strategies to reverse the impact of obesity on liver cancer progression is important due to the strong increased risk of liver cancer and its poor prognosis. Future translational research studies can be built from this pre-clinical foundational research.

## 1. Introduction

Hepatocellular carcinoma (HCC) is a common primary liver cancer, constituting about 75% of all liver cancer types and being one of the leading causes of cancer deaths internationally [1]. While treatment options and mortality rates have improved for HCC, the current 2-year survivability rate is 50%, which decreases to 10% after 5 years [1]. Males are 2-4 times more likely to develop HCC compared with females due to behavioral risk factors such as smoking and alcohol consumption, while Asian Americans, African Americans, and Hispanics have the highest rates based on ethnicity [2]. Early detection of HCC can result in effective treatments such as liver transplantation or surgery, leading to an improved prognosis over time [1]. Even so, most patients are diagnosed when HCC is in an advanced stage [1]. Chemotherapy is a common cancer treatment used to inhibit cancer cell viability and proliferation, with the most common agent being sorafenib [1]. While sorafenib’s mechanism of action is to block specific kinase pathways, it is often only able to increase survival by 7–10 months, indicating the need to develop other effective liver cancer treatment options [1].

Non-alcoholic steatohepatitis (NASH), alcoholic cirrhosis, hepatitis B and C, and non-alcoholic fatty liver disease (NAFLD) are major contributors to the development of HCC in the United States population [1]. Obesity, diabetes, metabolic syndrome, and a variety of other disease states increase the risk of the development and progression of HCC through exacerbation of NAFLD [3]. McGlynn et al. reported that the risk of developing HCC was 2.6-fold higher in obese patients with NAFLD compared with non-obese patients with NAFLD [3]. Obesity has been shown to be a strong underlying factor in the development of NASH and NAFLD, specifically through the upregulation of certain adipokines, subsequently leading to tumorigenesis [3]. Clinical data show a correlation between NAFLD and obesity, with the high prevalence of steatohepatitis in those with class I–II obesity being 65% and 85% in those with class III obesity [4]. According to the US Cancer Statistics database, 4.7% of cancer cases in men and 9.6% of cancer cases in women were due to excess body weight, with 51% of those cases being liver or gallbladder cancer [5]. Data have also shown that obesity is associated with a 13% risk of recurrence and poor outcomes in survivors [5]. Obesity is indicative of a high level of adipose tissue, which plays a role in the impairment of metabolic and hormonal control, thus leading to carcinogenesis [6]. While the obesity-cancer connection is multifactorial, research has shown that there are three primary mechanisms responsible for the increased risk of cancer in obese individuals [6]. For one, adipose tissue can increase the production of hormones and adipokines, which may be anti-apoptotic and pro-proliferative when excreted in excess concentrations by adipocytes [6]. The overproduction of these hormones, such as leptin, leads to proinflammatory environments with excess and sustained levels of obesity-associated hormones. However, when overexpressed, they can contribute to tumorigenesis through the activation of pro-proliferative and anti-apoptotic mechanisms by binding to its receptor and stimulating pro-tumorigenic cellular changes [6]. Furthermore, obesity is often associated with hyperinsulinemia, with high levels of insulin and insulin-like growth factor 1 (IGF-1), a pro-tumorigenic hormone, aiding in the development of cancer. In certain cases, this can lead to progression due to its pro-proliferative and anti-apoptotic characteristics [6,7]. While the hormone itself is not responsible for the appearance of cancer cells, it contributes to the uncontrolled growth and survival of tumor cells [7]. Those properties exacerbated by obesity, such as resistance to cell death, proliferative signaling, and cell immortality, have been deemed to be just a few of the hallmarks of cancer that are adversely affected by obesity.

Obesity is the primary cause of NAFLD development, a precursor to NASH, in about 50% of HCC patients, both of which are characterized by lipid accumulation and inflammation in the liver [8]. Prolonged lipid accumulation will subsequently lead to the progression of liver disease, evidenced by the presence of steatohepatitis and ballooning of the hepatocytes. NASH is the result of enlarged hepatocytes and an inflamed liver due to steatohepatitis, eventually leading to fibrosis, cirrhosis, and the development of liver cancer if untreated [8]. Rajesh et al. suggested that insulin resistance and a high-caloric intake, often associated with obesity, can lead to a buildup of adipocytes, thus producing a pro-inflammatory microenvironment where hepatic cancer cells are able to survive [9]. Chronic injury to the liver, such as cirrhosis exacerbated by NAFLD, can also result in an overexpression of nuclear factor-kB (NF-kB), a pro-tumorigenic transcription factor and inflammatory mediator associated with liver cancer [10]. Obesity-induced leptin and IGF-1 can activate NF-kB transcription, leading to an increase in cytokines [10,11]. The release of these pro-inflammatory cytokines, such as interleukin (IL)-6, interleukin-1β (IL-1β), and tumor necrosis factor alpha (TNF-α), leads to the perpetuation of an inflammatory response, resulting in increased activation of signaling pathways that are associated with the hallmarks of cancer, including survivability, proliferation, reactive oxygen species (ROS) production, and invasion into other tissues [7,12].

There is a plethora of mechanistic pathways involved in the relationship between obesity and HCC. Nicotinamide ribosyl transferase (NAMPT) is an enzyme that is highly expressed in the liver and is involved in the progression of tumors in obesogenic environments, having been shown to have increased concentrations in the context of obesity [13]. NAMPT has two derivatives with varying functions: extracellular NAMPT (eNAMPT) and intracellular NAMPT (iNAMPT). eNAMPT, also known as visfatin, is an adipokine with an important role, though its involvement in carcinogenesis is not fully understood [13]. Recent research has shown that eNAMPT overexpression has been associated with apoptotic resistance, cancer cell proliferation, and activation of the pro-inflammatory pathways which are associated with NAD biosynthesis [13]. iNAMPT is known to be the rate-limiting enzyme in the synthesis of NAD, an essential coenzyme involved in numerous redox reactions that progress into the production of the cell’s means of energy, ATP, which promotes a pro-growth environment in the context of obesity [13]. While the role of iNAMPT in HCC is a novel concept, overexpressed iNAMPT levels have been detected in many other cancer types, including prostate, colorectal, and gastric tumors [14]. iNAMPT has been shown to have anti-apoptotic and proliferative properties on tumor cells and is associated with increased metastasis and tumor growth [13,14]. Because iNAMPT and cellular energetics, a hallmark of cancer, play an important role in liver cancer progression, it has become a target for therapies focused on inhibiting its function in cancer cells [15,16]. FK866, an FDA-approved iNAMPT inhibitor, has been shown to have antitumor properties through repression of cancer cell growth, dephosphorylation of the Erk and Akt pathways, and apoptotic induction, which could play an important role in cancers that are dependent on these pathways.

The purpose of this study was to determine the contribution of NAD biosynthesis in obesity-induced liver cancer progression. We hypothesized that liver cancer cells exposed to an obese person’s serum will show suppressed viability and increased apoptotic ability when treated with the iNAMPT inhibitor FK866. We also hypothesized through using a syngeneic mouse model of liver cancer that obese mice injected with an iNAMPT inhibitor will show a decrease in tumor incidence and growth. For both, we demonstrate that inhibition of iNAMPT resulted in suppression of tumor growth in the context of obesity. Identifying pre-clinical strategies to reverse the impact of obesity on liver cancer progression is important due to the strong increased risk of liver cancer and its poor prognosis. Future translational research studies could build upon pre-clinical foundational research.

## 2. Materials and Methods

### 2.1. Cell Culture

Both human and mouse liver carcinoma cell lines (SNU-449 and Hepa 1-6, respectively) were purchased from the American Type Tissue Culture Collection (ATCC). SNU-449 cells were cultured in RPMI plus 10% fetal bovine serum (FBS), and both HepG2 and Hepa 1-6 cells were cultured in EMEM plus 10% FBS. All cells were maintained at 37 °C in a 5% (*v*/*v*) CO_2_ humidified incubator. Cells were exposed to one of five experimental conditions: 5% obese (OB) mouse serum, 5% OB serum + FK866 (10 nM), 5% control (CR) mouse serum, and 5% CR + FK866 (10 nM). FK866 (cat # HY-50876) was obtained from Medchemexpress USA (Monmouth Junction, NJ, USA). The times of exposure will be explained further in each assay described below.

### 2.2. Mouse Serum

Mouse serum from C57BL/6J mice was purchased from Jackson Laboratories (Bar Harbor, ME, USA). Post weaning, the mice were randomized to a high-fat diet (60% kcal from fat) (OB) or control diet (CR) and continued their respective diets for the remainder of the study. For the OB group, 12 mice sera were pooled at 14 weeks of age. For the CR group, 12 mice sera were pooled at 14 weeks of age. OB and CR sera were used for in vitro experiments at a concentration of 5%, according to previous research [17].

### 2.3. MTT Assay

To measure cell viability, an MTT assay was performed. HepG2, SNU-449, and Hepa 1-6 cells were seeded in a 96-well plate with 10,000 cells per well. The cells were incubated overnight in FBS. After 24 h, the cells were treated with the treatments stated above for 72 h. After 72 h, 20 µL of MTT reagent (5 mg/mL) was added to the well plate for 1.5 h. Then, the media along with the reagent was aspirated, and 100 µM of dimethyl sulfoxide (DMSO) was added to the well plate and shaken for 10 min at room temperature. Absorbance was measured at 540 nm using a Cytation 5 microplate reader (BioTek Instruments, Inc., Winooski, VT, USA).

### 2.4. Reactive Oxygen Species

The intracellular levels of reactive oxygen species (ROS) were assessed using a cell-permeable dichlorofluorescin diacetate (DCFH-DA) probe (ab113851) from Abcam (Cambridge, UK) according to the manufacturer’s procedure. SNU-449, HepG2, and Hepa 1-6 cells were seeded in a dark, clear-bottomed 96-well microplate. On day 2, the cells were exposed to the same experimental conditions described above. On day 3, the media were removed, and the cells were washed and incubated for 45 min with 25 μM DCFDA at 37 °C. The fluorescent intensity was measured at an excitation wavelength of 485 nm and an emission wavelength of 529 nm with a Cytation 5 microplate reader from BioTek Instruments, Inc. (Winooski, VT, USA).

### 2.5. Cytotoxicity

To assess cytotoxicity and early indicators of cell death, a lactate dehydrogenase (LDH) assay was used in the SNU-449, HepG2, and Hepa 1-6 cell lines according to the manufacturer’s instructions with Sigma’s Lactate Dehydrogenase Activity Assay Kit (MAK066-1KT) (MilliporeSigma, Burlington, MA, USA).

### 2.6. Invasion Assay

A Corning (Corning, NY, USA) BioCoat Matrigel Invasion Chamber (#354480) assay was utilized to assess the addition of FK866 and its ability to decrease the invasive capacity of the HepG2, SNU-449, and Hepa 1-6 cell lines when exposed to the treatments described above. The Matrigel chamber was hydrated in serum-free media for 2 h prior to seeding. After the chambers were rehydrated, cells were seeded on the upper side of the chamber at a concentration of 80,000 cells per well in a 24-well plate and treated with the conditions described above. The chemoattractant, 10% FBS, was added to the well below the chamber. After 48 h, the cells were stained with crystal violet, and images were captured using a Cytation 5 microplate reader from BioTek Instruments, Inc. (VT, USA). After imaging, the crystal violet stain was dissolved using a distain solution (methanol, acetic acid, and water), and absorbance was measured with the Cytation5 microplate reader.

### 2.7. Immunoblot

To measure protein expression differences, SNU-449, HepG2, and Hepa 1-6 cells were plated at a density of 4 × 10^5^ cells per well in a 6-well plate. After 24 h, the cells were starved of serum for 6 h. The cells were then exposed to their respective experimental conditions for 15 min. After treatment, the cells were harvested using lysis buffer (5 mL glycerol, 3.14 mL TRIS 1 M (pH: 6.8), 5 mL 10% SDS, and 36.86 mL ddH_2_O) and quantified using a Pierce BCA protein assay kit (Thermo Fisher Scientific, St. Louis, MO, USA). Then, 50 μg of protein lysate was electrophoresed through a 10% SDS PAGE gel. The proteins were then transferred to a nitrocellulose membrane. The membrane was blocked with 5% bovine serum albumin for 45 min. Protein lysates were subjected to immunodetection with rabbit anti-phospho-Erk, rabbit anti-total Erk, rabbit anti-phospho-Akt, and rabbit anti-total Akt. Images were acquired using a Fotodyne gel documentation system (Hartland, WI, USA). After measuring the phosphorylated proteins, blots were stripped with Restore Plus western blot stripping buffer before the total protein levels were measured. ImageJ version 1.54p was used to quantify the protein levels.

### 2.8. Animal Model and Study Design

Twenty-one 8-week-old C57BL/6J male mice were ordered from Jackson Laboratories (Bar Harbor, ME, USA). Post weaning, 10 mice were randomized to a high-fat diet (ad libitum) that induces obesity (60% kcal from fat; D12492, referred to as DIO), and 11 mice were randomized to a control diet (10% kcal from fat; D12450B, referred to as CR). Both remained on these diets for the duration of the study. The mice received new food 2 times per week. The mice were weighed weekly to monitor weight gain. At 21 weeks of age, all mice were injected with 500,000 Hepa 1-6 liver cancer cells suspended in PBS for a total volume of 100 μL. At 23 weeks of age, FK866 was administered at a dose of 30 mg/kg dissolved in DMSO and phosphate buffered saline (PBS) for 3 days over 2 weeks. The DIO and CR mice were injected with a vehicle control consisting of DMSO and PBS at the same volume as the inhibitor. The concentration of FK866 was based on previous cancer mouse models [18,19]. The study was designed with a statistical power of 90% and a type 1 error of 5%. The sample size was determined by two primary outcomes of tumor and weight change, with a 50% reduction in tumor size and 30% increase in body weight when compared with their controls. The calculated minimum sample sizes were 2 mice per group for tumors and 3 mice for weight.

### 2.9. Tumor Injection, Incidence, and Growth

For each cancer cell injection, a 500,000 Hepa 1-6 tumor liver cancer cell suspension was added to PBS and 25% matrigel BME Type 3 (Cat # 3632-005-02) R&D Systems (Minneapolis, MN, USA). The ratio of the cell suspension to PBS was 1:1 with a maximum volume of 250 µL. The cell suspension was injected subcutaneously on the flank of the C56BL/6 mouse with a 25 g needle. The mice were monitored daily until a palpable tumor was detected. As a baseline, the mice were weighed on the day of implantation. Once a palpable tumor was detected, the mice were monitored daily. Twenty-four hours post cancer cell injection, the mice were monitored for palpable tumors. Tumor incidence was determined by counting the number of palpable tumors in each experimental group.

### 2.10. Statistical Analysis

All in vitro experiments were replicated at least 3 times to ensure validity. GraphPad Prism version 10.5.0 (Boston, MA, USA) was used to analyze the results via one-way ANOVA with a Tukey’s post hoc test. Results containing *p* < 0.05 were considered statistically significant.

## 3. Results

### 3.1. NAD Inhibition Reduced Serum-Induced Viability

**Figure 1:**
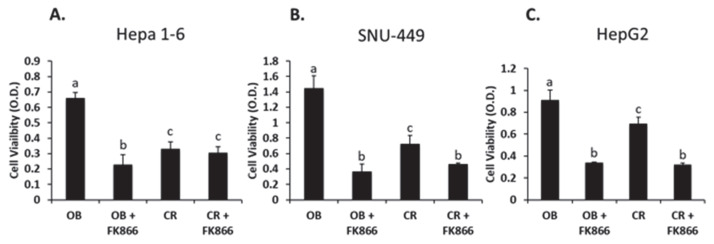
MTT assay used to assess cell viability. Viability was assessed via MTT dye conversion. Hepa 1-6 (**A**), SNU-449 (**B**), or HepG2 (**C**) cells were exposed to the treatments for 72 h. OB = obese sera, FK866 = iNAMPT inhibitor, and CR = control sera. Data shown represent the average of at least three independent experiments. Results were compared using ANOVA followed by Tukey’s post hoc test. Different letters indicate significant differences between experimental conditions; *p* < 0.05.

Cell proliferation is important to assess in cancer progression, as excessive cell viability can lead to dysregulation in cellular homeostasis, thus leading to an increase in tumorigenesis [7]. All three cell lines were exposed to obese (OB) and control (CR) sera with or without FK866 to assess viability through an MTT assay (Figure 1). When comparing the OB and control sera, Hepa 1-6, SNU-449, and HepG2 were shown to have increases in viability in the OB serum of 98%, 98%, and 31%, respectively (*p* < 0.05). The addition of FK866, an NAD inhibitor, to the OB serum suppressed cell viability by 69% in the Hepa 1-6 cell line, 71.4% in the SNU-449 cell line, and 46% in the HepG2 cell line (*p* < 0.05). The addition of FK866 to CR in all cell lines also showed a decrease in cell viability, with significant changes observed in SNU-499 and HepG2 (*p* < 0.05). These data suggest that FK866 does reduce obesity-induced viability.

### 3.2. Addition of FK866 Decreased Obesity-Induced ROS Production

**Figure 2:**
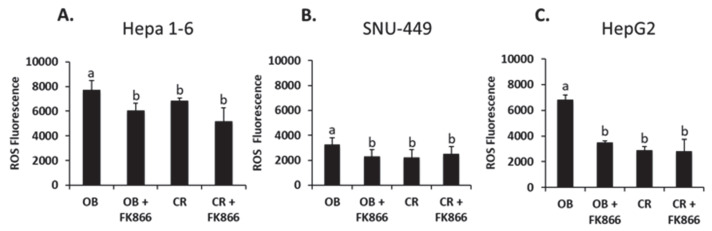
ROS fluorescence analysis in liver cancer cells. Hepa 1-6 (**A**), SNU-449 (**B**), or HepG2 (**C**) cells were exposed to the treatment for 24 h. After 24 h, the cells were labeled with DCFDA (20 μM) and then analyzed on a fluorescent plate reader. OB = obese sera, FK866 = iNAMPT inhibitor, and CR = control sera. Data shown represent the average of at least three independent experiments. Results were compared using ANOVA followed by Tukey’s post hoc test. Different letters indicate significant differences between experimental conditions; *p* < 0.05.

In areas of prolific and chronic inflammation, macrophages and neutrophils will produce ROS in order to destroy cancer cells [20]. Using a cell-permeable (DCFH-DA) probe, liver cancer cell lines were exposed to the previously mentioned treatments to assess ROS production through fluorescence. As shown in Figure 2, Hepa 1-6 and SNU-449 were shown to have increases in ROS production when exposed to OB serum of 27% and 41%, respectively, in comparison with the CR serum (*p* < 0.05). HepG2 cells exposed to the OB serum doubled ROS production compared with the CR serum (*p* < 0.05). In HepG2, the addition of FK866 to OB serum decreased ROS production by 96% when compared with OB serum alone (*p* < 0.05). A similar decrease in ROS production can also be seen in the Hepa 1-6 and SNU-499 cell lines (*p* < 0.05). No significant differences were observed when comparing CR serum to the addition of FK866 in CR serum in all three cell lines. These data suggest that obesity-induced ROS production is inhibited by the addition of FK866.

### 3.3. NAD Inhibition Promoted LDH Secretion

**Figure 3:**
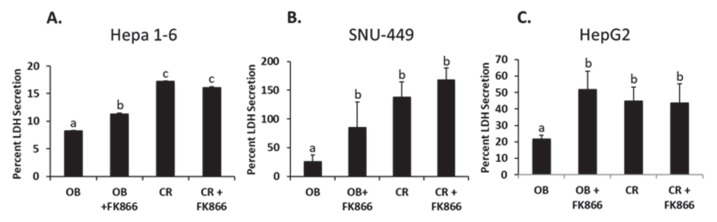
LDH secretion by liver cancer cells exposed to serum. Hepa 1-6 (**A**), SNU-449 (**B**), or HepG2 (**C**) were exposed to treatments in serum free media. OB = obese sera, FK866 = iNAMPT inhibitor, and CR = control sera. Cells remained in treatment for 24 h. Thereafter, an Invitrogen LDH Cytotoxicity Kit was used to assess LDH secretion in the cells following treatments. Different letters indicate significant differences between experimental conditions; *p* < 0.05.

Cytotoxicity assessment via LDH secretion was used to assess the apoptotic ability of tumor cells when exposed to the inhibitor [21]. The OB serum decreased LDH secretion by 52% in Hepa 1-6, 60% in SNU-449, and 51% in HepG2 when compared with the CR serum (*p* < 0.05). The addition of FK866 to the OB serum showed an additional 18% decrease in Hepa 1-6, 27% decrease in SNU-449, and 57.7% decrease in HepG2 (*p* < 0.05). No significant differences in LDH secretion were observed between the CR serum and the addition of FK866 to the CR serum in any of the three cell lines (Figure 3). These data suggest that LDH secretion is reduced and cell death increases with the addition of an NAD inhibitor in an obesogenic environment.

### 3.4. NAD Inhibition Reduced Obesity-Induced Invasive Potential

**Figure 4:**
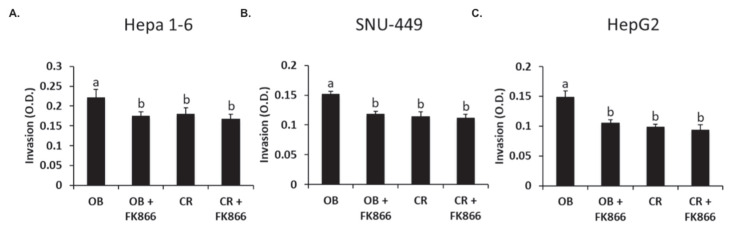
Invasion capacity of liver cancer cells. Hepa 1-6 (**A**), SNU-449 (**B**), or HepG2 (**C**) liver cancer cells were seeded in BD Biocoat Matrigel Chambers. OB, OB + FK866, CR, and CR + FK866 were added to the top of the chambers, and FBS was added to the bottom chamber. After 48 h, cells were fixed, stained, and counted. Data shown represent the average of at least three independent experiments. Different letters indicate significant differences between experimental conditions; *p* < 0.05.

Cellular invasion is a metastatic process where malignant tumor cells spread to surrounding tissues. The invasion assay was used to determine the efficacy of inhibiting NAD synthesis to reduce the invasive potential of cancer cells exposed to sera. As shown in Figure 4, the invasive capacity was increased by 22% in Hepa 1-6, 32% in SNU-449, and 51% in HepG2 when cells were exposed to the OB serum (*p* < 0.05). Furthermore, the addition of FK866 to the OB serum reduced the invasive capacity in Hepa 1-6, SNU-449, and HepG2 by 26%, 28%, and 41% respectively (*p* < 0.05). No significant differences were observed with the addition of FK866 to the CR serum. These data suggest that FK866 decreases the invasive capacity of cancer cells by inhibiting a pro-growth enzyme.

### 3.5. Differential Effects of NAD Inhibition on Kinase Signaling

**Figure 5:**
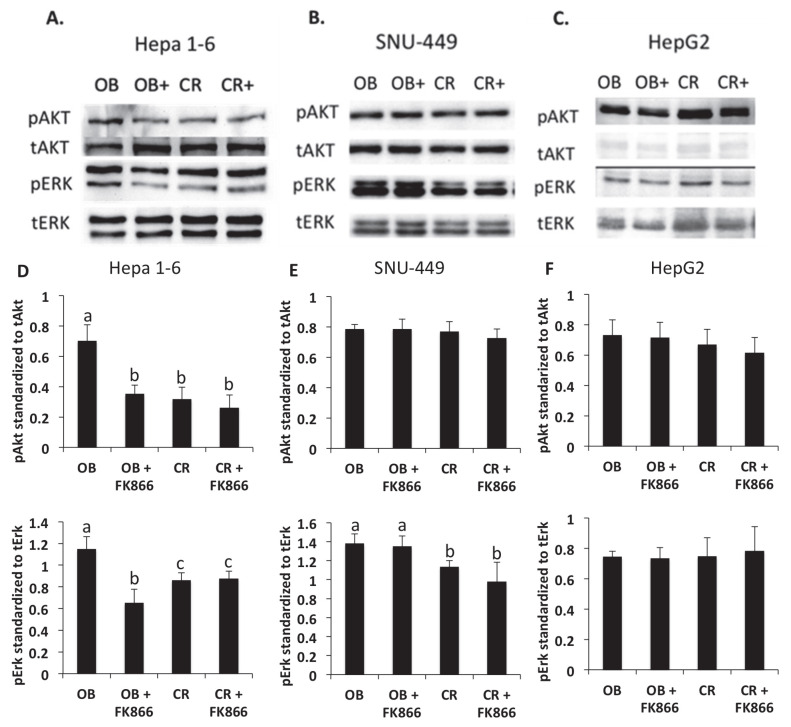
Western blot analysis of kinase phosphorylation in liver cancer cells. Hepa 1-6 (**A**), SNU-449 (**B**), or HepG2 (**C**) cells were seeded at 250,000 cells/well for 24 h. Cells were serum-starved for 24 h and then treated in SFM with 5% OB sera, 5% OB sera + FK866 (iNAMPT inhibitor) 10 nM, 5% CR sera, and 5% CR sera + FK866 10 nM for 1 h. Pixel density was measured to quantify protein levels using imageJ. (**D**) Hepa 1-6 pixel density of phosphorylated Akt standardized to total Akt and phosphorylated Erk standardized to total Erk. (**E**) SNU–449 pixel density of phosphorylated Akt standardized to total Akt and phosphorylated Erk standardized to total Erk. (**F**) HepG2 pixel density of phosphorylated Akt standardized to total Akt and phosphorylated Erk standardized to total Erk. Data shown represent the average of at least three independent experiments. Different letters indicate significant differences between experimental conditions; *p* < 0.05.

Activation of kinase signaling pathways, such as Erk and Akt, has been shown to be associated with pro-tumorigenic characteristics, such as tumor growth, proliferation, and invasion into other tissues [22]. In Figure 5, Akt and Erk kinase signaling pathways were assessed via immunoblot to investigate the effect of obesity and the impact of FK866 on protein levels. In the Hepa 1-6 cell line, pAkt and pErk were shown to be upregulated in the OB serum and reduced with the addition of FK866 (*p* < 0.05). The SNU-449 cell line was shown to have an increase in pErk in the presence of obesity but with different effects with the addition of FK866 in CR + FK866. Lastly, differences were not observed between the OB and CR groups or the addition of FK866 to either group in the HepG2 cell line.

### 3.6. High-Fat Diet Increased Body Weight, Tumor Incidence, and Tumor Volume

**Figure 6:**
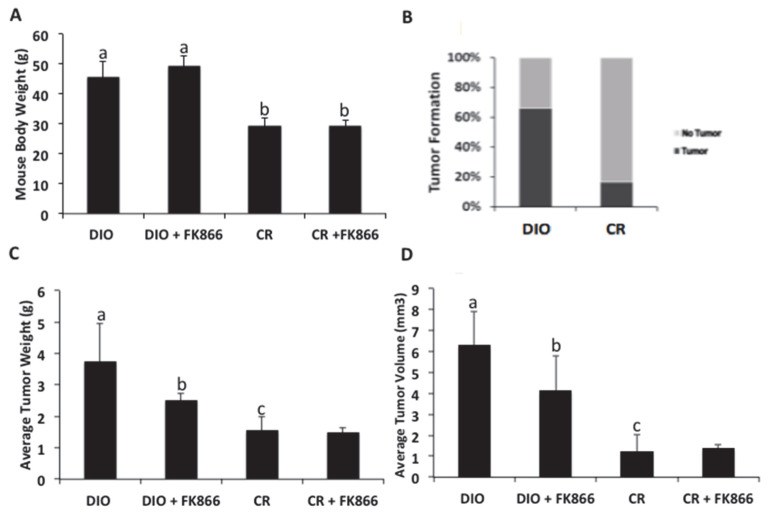
Effects of high-fat diet and FK866 inhibitor on C57/BL6 mice. At 21 weeks, C57/BL6 mice were injected with 500,000 Hepa 1-6 liver cancer cells, and palpable tumors were detected at 23 weeks. (**A**) Mice were randomized to high-fat diet (DIO) or control (CR) diet for 25 weeks, and final body weights were measured. (**B**) Tumor incidence was confirmed at the end of the study, with percentages determined from mice randomized to either the DIO or CR group. (**C**) Tumor weight was measured at the end of the study for each experimental group: DIO (*n* = 3), DIO + FK866 (*n* = 4), CR (*n* = 2), and CR + FK866 (*n* = 1). (**D**) Tumor volume was measured for all mice using the following formula to calculate tumor volume: tumor volume = d2 × D/2. Here, d and D represent the shortest and longest diameter in mm, respectively. Different letters indicate significant differences between experimental conditions; *p* < 0.05.

Mouse body weights and tumor formations were collected. Body weight was used to assess the effect of a high-fat diet on weight gain through increased adiposity in the mice. The mice on a high-fat diet had a 54% increase in body weight, a significant difference in weight compared with those on the control diet (*p* < 0.05), as shown in Figure 1A. As anticipated, the mice fed a high-fat diet demonstrated increased body weights and tumor incidence. Tumor formation was determined by daily palpation of tumor injection sites. In the DIO mice, 66% (seven out of nine mice) had the presence of a tumor, while only 20% of the CR mice (one out of five mice) were determined to have tumors (Figure 6B). Since the mice were randomized, for transparency, three of the five DIO mice had tumors, and four of the five DIO + FK866 mice developed tumors before treatment, while two of the six CR mice and one of the five CR + FK866 mice developed tumors. The DIO mice developed tumors that weighed twice those of the CR mice (*p* < 0.05), and the addition of FK866 decreased the tumor weights for the DIO mice by 39% (Figure 6C). Similarly, FK866 reduced the tumor volume in the DIO mice by 57%. The DIO mice tumor volumes were significantly higher compared with the CR mice (*p* < 0.05) (Figure 6D). Lastly, adding FK866 to the CR mice did not result in tumor weight or volume changes compared with the CR mice without treatment, thereby demonstrating DIO-specific efficacy in affecting tumor weight and volume.

## 4. Discussion

Excess adiposity, as a result of obesity, produces pro-inflammatory adipokines and cytokines that support tumorigenesis by increasing proliferation and enhancing the biomolecules necessary for growth. One adipokine, NAMPT, and its different derivatives (eNAMPT and iNAMPT) are key targets for novel therapeutics. iNAMPT has been previously established as being overexpressed in other types of cancers and positively correlated to high levels of adiposity. As obesity increases, iNAMPT levels also increase as a result, leading to the upregulation of harmful and pro-tumorigenic changes in the liver, such as increased proliferation, ROS production, invasion, and survivability [14]. The connection between iNAMPT in the presence of obesity and its role in tumorigenesis makes it an important target of inhibition in order to possibly halt cancer progression.

Studies have shown that obese phenotype serum increases cell proliferation compared with serum from a lean phenotype [23]. The data in this study follow a similar trend, where the liver cancer cell lines treated with obese phenotype serum resulted in increased proliferation compared with the control group, thereby demonstrating that obesity promotes cell growth. On the other hand, other studies involving the use of FK866 as an NAMPT inhibitor have shown that FK866 reduces the cell viability of other hepatic cancer cell lines in high doses (5–40 nM) [24,25]. According to the data from this study, FK866 treatment in all three cell lines resulted in a similar trend, with cell proliferation being decreased with the addition of the NAMPT inhibitor. This suggests that blocking NAD synthesis with FK866 has a significant effect on reducing liver cancer cell viability in the context of obesity.

Oxidative stress because of ROS production from inflammation and metabolic processes is common in many disease states, including cancer and increased adiposity [26]. The intracellular destruction that follows the production of ROS creates an ideal environment for cancer to progress. The presence of obesity has been shown to be a pathological process that may induce oxidative stress due to the excretion of pro-inflammatory cytokines from adipocytes such as IL-6 and TNF-α. When inflammation is exacerbated by obesity due to secretion of adipocytokines, mitochondrial function can be altered, which leads to increased production of ROS [27]. One study demonstrated a reversal in ROS production with the introduction of FK866 to cells treated with hydrogen peroxide [28]. The data from the current study show that the cells treated with obese phenotype serum had a significant increase in ROS production compared with the control, whereas the addition of FK866 to the obese group decreased ROS in all three cell lines. The high levels of adiposity associated with obesity create a pro-inflammatory environment that degrades metabolic processes, possibly leading to the accumulation of ROS and thus perpetuating inflammation. Previous research on ROS levels in tumorigenesis have shown that ROS, as well as other molecules, are responsible for the activation of signaling cascades that lead to proliferation, invasion, suppression of apoptosis, and thus the formation of larger tumors [29]. A larger tumor size can have an effect on prognosis and treatment outcomes in those with HCC. Collectively, the combined in vitro and in vivo data from the current study also suggest that the inhibition of NAD biosynthesis via FK866 suppresses ROS production and may decrease tumor growth. However, this study did not test the mechanism by which ROS suppression inhibits tumor growth. In addition, while the murine Hepa 1-6 and human HepG2 liver cancer cell lines had significant decreases in ROS production with the addition of FK866, the same pattern was not as robust in SNU-449. This difference may be due to metabolic differences in the enzymatic pathways and genetic background between the liver cell lines used in this study. Because ROS is a byproduct of cellular metabolism, future directions may involve investigating the mechanisms by which NAD inhibition slows the release of ROS in order to decrease the overall production of ROS in an obesogenic environment.

Cell survivability and the avoidance of cell death are two of the hallmarks of cancer and have become popular points of interest for treatment [7]. LDH is commonly excreted by cells in the early stages of cell death, also known as apoptosis [30]. In other words, LDH secretion is a precursor event prior to initiating the steps leading to cell death. One study found that leptin, an adipocytokine known to have increased levels during cancer development, impaired natural killer cell cytotoxicity. While the methods in this study assessed cytotoxicity through the secretion of LDH, the data remained consistent, as obesity decreased LDH secretion and thus cytotoxicity. In another study, it was shown that treating glioblastoma cells with FK866 increased LDH’s release, suggesting that more cells were undergoing apoptosis when NAD biosynthesis was inhibited [30]. The findings of this study followed a similar pattern, with OB by itself decreasing LDH secretion and the addition of FK866 increasing secretion in all three cell lines. The decrease in LDH indicates less cytotoxicity, meaning the cells were not undergoing apoptosis. This may suggest that the presence of obesity protects cells from cell death. Though the exact mechanism is unknown, it may be associated with iNAMPT. Despite this, the addition of FK866 shows that there is a key pathway that can be blocked with the inhibition of NAD biosynthesis, thus sensitizing cells to apoptosis. These data are also supported by the cell viability assay used in this study, where viability was shown to have increased in the presence of obesity yet decreased with the addition of FK866. To extend these findings, future studies may investigate analyzing the specific mechanisms behind cytokines released by adipocytes and its protective ability against cell death. On the other hand, future studies may also investigate strategies to sensitize cells to apoptosis in order to reverse the protective ability of obesity-associated tumorigenic proteins.

Cellular invasion is a critical step in metastasis, which is the process of cancer cells spreading to other tissues and causing destruction to other body systems. One study shows that the addition of adipocytes to prostate cancer cells increased cell invasion significantly compared with cells that were not treated with adipocytes [31]. In another study, when MHCC97-H cells were treated with the NAMPT inhibitor FK866, the cells were shown to be significantly less invasive than those without the inhibitor [24]. Thus, previous research aligns with the results observed in this study, which showed that all cell lines exposed to OB serum had an increase in invasion compared with the control, whereas the addition of FK866 to the OB groups significantly decreased invasion in all three lines. These results suggest that the presence of obesity-associated proteins such as hormones and cytokines may increase the invasive capacity of tumor cells but can be decreased via the inhibition of NAD biosynthesis. In addition to invasion, future studies could investigate the role of obesity-associated hormones in enhancing the metastatic cascade and the role of visfatin in promoting the epithelial to mesenchymal transition, which is a key process in the early stages of invasion.

Erk and Akt are key signaling pathways that are known to be upregulated in both obesity and cancer. In one study, both kinases were shown to be increased in the presence of adipocytes, confirming the results found in this study, where pAkt and pErk protein levels were increased in the presence of obesity-associated hormones and cytokines [29]. Studies have shown that the upregulation of these kinase pathways has been implicated in obesity-induced cancer progression through stimulating signal cascades that increase cell proliferation, growth, and metabolism [29]. Another study demonstrated the role that IGF-1, a hormone elevated in obesity, plays in activating downstream Erk and Akt signaling pathways, showing that upregulation of the pathways via IGF-1 led to the promotion of cancer cell invasion and proliferation [30]. One study demonstrated that the inhibition of IGF-1 signaling reduced Akt and Erk phosphorylation and thus slowed cancer proliferation, though this was correlative [32]. The results of this study were shown to be consistent with other findings, where inhibition of NAD via FK866 reduced the Akt protein levels in Hepa 1-6. However, the results were not consistent among all three cell lines, indicating that each cell line may have altered pathways associated with tumorigenesis. Obesity-associated hormones activate kinases that can promote downstream transcription factors, such as NF-kB, which leads to the production of more cytokines and proteins that affect invasion and proliferation. The mechanism by which FK866 inhibits the signal mediating these physiological outcomes needs to be explored further. Furthermore, mechanistic studies, a limitation of this study, may also investigate the mechanisms by which NAD inhibition mediates Erk and Akt signaling differently in liver cancer cell lines through proof of principle studies, such as gene silencing or knockdown models.

A high-fat diet has been previously shown to alter the metabolic ability of liver cells, eventually leading to liver cancer progression through increased ROS production and DNA damage. Feeding mice a high-fat diet has been shown to accelerate liver cancer development. This aligns with the results found in this study, where approximately 70% of the mice fed a high-fat diet developed tumors, while only 1% of the mice on a control diet developed tumors. An obesogenic environment promotes tumor growth, evidenced by increased weights and volumes. Importantly, NAD biosynthesis and cellular energetics play a role in obesity-induced growth. This may be a beneficial target, as suppressing this enzyme decreased these effects. These data suggest that an obese environment plays a significant role in the development and sustainability of liver cancer growth.

Elevated expression of the NAMPT enzyme and extracellular NAMPT has been found in the tumor tissues of HCC patients [33,34]. Both the enzyme and ligand function of NAMPT play an important role in supplying energy needs within cells. This is relevant to cancer as deregulated cellular energetics is a hallmark of cancer. Upregulated NAMPT in the scavenger pathway is a central contributor to the NAD+ pool, which can supply cancer cells with the ability to meet increased energy needs. However, NAMPT has different roles in various inflammatory-associated diseases related to infection, diabetes, and anti-microbial responses [34,35,36,37,38,39,40,41,42]. Therefore, when considering the suppression of NAMPT as an intervention, it is essential to consider the mechanistic context and tissue-specific effects.

While FK866 has demonstrated promising anti-cancer effects through iNAMPT inhibition and subsequent NAD+ depletion, several studies have reported potential side effects that warrant caution. In a Phase I clinical trial involving cancer patients with advanced solid tumors refractory to standard therapies, thrombocytopenia was identified as a dose-limiting toxicity at higher doses, along with fatigue, nausea, and decreased lymphocyte counts [18]. Although the authors reported patient side effects to range between mild and moderate [18], pre-clinical data have identified additional side effects. In healthy cardiomyocytes, FK866 treatment impaired mitochondrial metabolism and stress responses, suggesting potential cardiovascular risks in non-cancerous tissues [43]. In HCC cells, higher concentrations of FK866 (20–40 nM) were noted to potentially harm normal cells, prompting the authors to utilize lower FK866 doses (0–10 nM) in their research and study design, which is in alignment with the dosage utilized in this study [24]. Interestingly, FK866 treatment increased ROS activity in cholangiocarcinoma cells [44], which contrasts with our findings, where FK866 reduced ROS, suggesting that the effects of FK866 on oxidative stress may be cell type-, dosage-, and context-specific. As with many chemotherapeutic or adjuvant interventions, the adverse effects on non-cancerous tissues should be considered when designing future studies. Collectively, these findings demonstrate that FK866 holds therapeutic promise.

The data found in this study contribute to understanding the impact that obesity has on tumorigenesis and possible interventions that may block obesity-induced progression. Still, further analyses need to be performed to understand the role of energy production and cancer development, such as looking at the NAD/NADH ratio to assess FK866’s efficacy in blocking NAD synthesis, MMP-9, and inflammatory markers in tissue and completing serum analysis of the current mouse model. One strength of this study is the novel strategy to break the obesity-liver cancer link through the exploitation of cellular energetics, specifically blocking NAD biosynthesis. Currently, cancer therapeutics primarily focus on inhibition angiogenesis, signal interruption to inhibit cell growth, and promoting cancer cell death through apoptotic induction or through the delivery of toxins, radiation, and other cell-killing substances. Another strength of this study is the use of pooled obese sera to look at the collective effects of combined hormones, though this may also be considered a limitation since it is difficult to assess the specificity of what hormones or enzymes in sera promote pro-tumorigenic effects. In future studies, it may be useful to determine the contribution of individual hormones through the addition of a neutralizing antibody to target specific proteins upregulated in obesity. Finally, while the primary aim of this study was to look at the efficacy of FK866 in an obesity-indued liver cancer model, the mechanistic actions behind FK866 should be elucidated to improve understanding of its role in NAD inhibition.

## 5. Conclusions

In conclusion, the purpose of this study was to determine the role obesity plays in liver cancer progression and how targeting cellular energetics through NAD inhibition can lessen the impact of obesity on carcinogenesis. This study found that the presence of obesity significantly increases cancer cell proliferation, invasion, and ROS production and decreases cytotoxicity, all of which are hallmarks of cancer. On the other hand, it was found that NAD inhibition via FK866 was able to reverse obesity-induced hallmarks, thus signifying its efficacy as a potential intervention for liver cancer in an obesogenic environment.

## Data Availability

The datasets presented in this article are not readily available because the data are part of an ongoing study. Requests to access the datasets should be directed to the corresponding author.
